# Complete Genome Sequence of Escherichia coli Podophage Peacock

**DOI:** 10.1128/MRA.01056-19

**Published:** 2019-09-26

**Authors:** Erin Ruhlman, Ryan Bockoven, Russell Moreland, Mei Liu, Jolene Ramsey

**Affiliations:** a Center for Phage Technology, Texas A&M University, College Station, Texas, USA; Loyola University Chicago

## Abstract

Escherichia coli is typically a commensal bacterium of the mammalian intestinal tract. Here, the isolation and annotation of the 39,233-bp T7-like E. coli podophage Peacock genome are described.

## ANNOUNCEMENT

Escherichia coli is a Gram-negative, rod-shaped bacterium found in the mammalian intestinal tract. It has been widely studied due to its high growth rate and genetic plasticity (1). Although E. coli is typically a commensal organism, pathogenic strains can arise, for instance, through the acquisition of virulence factors through horizontal gene transfer (2). Antibiotics remain the standard of treatment even though many strains have become resistant (3). Investigating E. coli bacteriophages might provide insight into potential alternative treatments.

Phage Peacock was isolated from filtered (0.2-μm pore size) wastewater treatment plant influent samples collected in College Station, TX, using E. coli 4s as a host (4). The host was cultured aerobically on lysogeny broth/agar at 37°C with the soft agar overlay method, and genomic DNA was extracted from this phage, as previously described, using the shotgun library preparation modification to the Promega Wizard DNA clean-up system (5, 6). A Peacock DNA library was prepared with the TruSeq Nano low-throughput kit and sequenced on an Illumina MiSeq platform with paired-end 250-bp reads using V2 500-cycle chemistry. The 570,679 total sequence reads from the index containing the phage genome were evaluated using FastQC (http://www.bioinformatics.babraham.ac.uk/projects/fastqc/) and trimmed using the FASTX-Toolkit v0.0.14 (http://hannonlab.cshl.edu/fastx_toolkit/). The Peacock genome was assembled in a single raw contig with SPAdes v3.5.0 and had 513.9-fold coverage (7). PCR across the contig ends (forward primer 5′-TGTATGGGTATCATCGGGACA-3′ and reverse primer 5′-CCTCCTTGGACTTAGGGTCATA-3′) and Sanger sequencing were used to manually verify that the correct and complete phage sequence was present in the assembly. All annotation tools described below are in Galaxy and Web Apollo, hosted by the Center for Phage Technology (https://cpt.tamu.edu/galaxy-pub) (8, 9). Gene calling for the genome was completed using Glimmer v3.0 (10) and MetaGeneAnnotator v1.0 (11). No tRNAs were detected using ARAGORN v2.36 (12). Gene functions were predicted using InterProScan v5.33-72 (13), BLAST v2.2.31 at a 0.001 maximum expectation value (14), and TMHMM v2.0 (15) at default settings. The NCBI nonredundant and UniProtKB Swiss-Prot/TrEMBL databases were used in BLAST functional analysis (16). LipoP v1.0 was used to assess lipobox presence in spanins (17). Rho-independent termination sites were annotated from TransTermHP v2.09 (18). Genome-wide DNA sequence similarity was calculated using progressiveMauve v2.4.0 (19). Unless otherwise stated, all tools were executed using default parameters. To determine phage morphology, samples were negatively stained with 2% (wt/vol) uranyl acetate and viewed using transmission electron microscopy at the Texas A&M Microscopy and Imaging Center (20).

The 39,233-bp genome of the podophage Peacock has a G+C composition of 50.1% and a 92.4% coding density. Of the 55 protein-coding genes identified, 30 functions were predicted, and 27 of those had direct BLASTp hits to phage T7 (GenBank accession number NC_001604). Using PhageTerm, Peacock was predicted to have 179-bp direct terminal repeats (21). Peacock has 85% nucleotide sequence identity with *Escherichia* phage Vec13 (GenBank accession number MH400309), a T7-like phage, and shares 47 proteins with this phage. An overcome classical restriction (ocr) protein (NCBI accession number QEG09667), known to inhibit type I DNA restriction enzymes by mimicking a B-form DNA structure, was also identified (22).

### Data availability.

The genome sequence and associated data for phage Peacock were deposited under GenBank accession number MK903279, BioProject accession number PRJNA222858, SRA accession number SRR8892143, and BioSample accession number SAMN11408679.
